# Frailty is associated with the epigenetic clock but not with telomere length in a German cohort

**DOI:** 10.1186/s13148-016-0186-5

**Published:** 2016-02-26

**Authors:** Lutz Philipp Breitling, Kai-Uwe Saum, Laura Perna, Ben Schöttker, Bernd Holleczek, Hermann Brenner

**Affiliations:** German Cancer Research Center (DKFZ), Division of Clinical Epidemiology and Aging Research, 69120 Heidelberg, Germany; Epidemiological Cancer Registry of Saarland, Saarbrücken, Germany; Network Aging Research, University of Heidelberg, Heidelberg, Germany

**Keywords:** Telomere length, CpG methylation, Epigenetic age acceleration, Frailty index, Cross-sectional study, General population

## Abstract

**Background:**

The epigenetic clock, in particular epigenetic pre-aging quantified by the so-called DNA methylation age acceleration, has recently been suggested to closely correlate with a variety of disease phenotypes. There remains a dearth of data, however, on its association with telomere length and frailty, which can be considered major correlates of age on the genomic and clinical level, respectively.

**Results:**

In this cross-sectional observational study on altogether 1820 subjects from two subsets (*n* = 969 and *n* = 851; mean ± standard deviation age 62.1 ± 6.5 and 63.0 ± 6.7 years, respectively) of the ESTHER cohort study of the elderly general population in Germany, DNA methylation age was calculated based on a 353 loci predictor previously developed in a large meta-study, and the difference-based epigenetic age acceleration was calculated as predicted methylation age minus chronological age. No correlation of epigenetic age acceleration with telomere length was found in our study (*p* = 0.63). However, there was an association of DNA methylation age acceleration with a comprehensive frailty measure, such that the accumulated deficits significantly increased with increasing age acceleration. Quantitatively, about half an additional deficit was added per 6 years of methylation age acceleration (*p* = 0.0004). This association was independent from age, sex, and estimated leukocyte distribution, as well as from a variety of other confounding variables considered.

**Conclusions:**

The results of the present study suggest that epigenetic age acceleration is correlated with clinically relevant aging-related phenotypes through pathways unrelated to cellular senescence as assessed by telomere length. Innovative approaches like Mendelian randomization will be needed to elucidate whether epigenetic age acceleration indeed plays a causal role for the development of clinical phenotypes.

**Electronic supplementary material:**

The online version of this article (doi:10.1186/s13148-016-0186-5) contains supplementary material, which is available to authorized users.

## Background

DNA methylation patterns are known to change with chronological age, and multiple CpG sites with replicable associations with age have been identified [1]. Based on regression coefficients estimated from a large number of datasets, an individual’s chronological age can be predicted from DNA methylation data with high accuracy [2]. The difference between the thus predicted methylation age (DNAm age) and the chronological age has been termed “age acceleration” and, intriguingly, has been found to be a substantially heritable trait in twin study datasets that furthermore shows plausible associations with several phenotypes across studies and tissues [2].

Frailty describes a clinical syndrome characterized by a depletion of physical and cognitive resilience and reserves, commonly associated with an accumulation of functional deficits [3, 4]. Frailty has received growing attention in recent years, due to pronounced associations with longevity and other aging-related phenotypes and the corresponding perception that frailty measures reflect an individual’s clinically relevant biological age [4, 5]. Stable intra-individual differences in biological aging and accruing frailty exist [6], and DNA methylation patterns might play a role in this phenomenon [7].

Telomere length (TL) has been suggested to reflect an individual’s biological age at the genomic DNA level, and associations of measures of TL with various aging- and frailty-related phenotypes, such as sarcopenia [8] and bone loss [9], have been reported. Whereas epigenetic age acceleration has been shown to be associated with various cancer phenotypes [2], its correlation with TL apparently has not been investigated to date, and seemingly only one study has addressed age acceleration and frailty [10]. In a rare study analyzing both TL and DNAm age, the development of symptoms of post-traumatic stress syndrome was associated with both variables—in plausibly opposite, yet altogether unexpected directions—but their mutual correlation apparently was not investigated [11].

A better understanding of the interplay of epigenetic and genomic correlates of age as determinants of clinical frailty could help to elucidate novel pathways to healthy aging and longevity. In the present study, the correlation of epigenetic age acceleration with TL and frailty was investigated in two large subsets of a general population sample of community-dwelling older adults in Germany. In light of inconclusive previous findings on associations of TL with frailty [12], interaction analyses were conducted to evaluate whether DNAm age alters the association of TL with frailty.

## Results

### Description of study population and main variables

Major characteristics of the study populations are shown in Table 1. The two ESTHER subsets analyzed in the present work—dataset 1 consisting of 1000 consecutively recruited participants of the ESTHER cohort and dataset 2 originating from a case-cohort design (see “Methods” section for details)—resembled one another closely, although some of the slight differences between these two very large subsets were statistically significant. The mean age was 62.1 and 63.0 years, respectively. Methylation age and difference-based epigenetic age acceleration appeared somewhat higher in dataset 2, in which relative telomere length also tended to be lower. There was no difference with respect to the frailty index. Current smoking was prevalent in one fifth of participants in both datasets, and harmful alcohol consumption was reported by less than 10 % of participants. Histograms of the main analysis variables are shown in Additional file 1: Figure S1 and suggested some skewness of frailty only, whereas DNAm age acceleration and relative telomere length closely followed a normal distribution.

**Table 1:** Description of two subsets of the ESTHER study, an epidemiological study of the elderly general population in Germany

Characteristic		Dataset 1	Dataset 2	*p* ^a^
Total	*n*	969	851	
Age in years	μ (SD)	62.1 (6.5)	63.0 (6.7)	0.0078
Methylation age in years	μ (SD)	61.7 (7.1)	64.6 (7.7)	<0.0001
Age acceleration in years^b^	μ (SD)	−0.5 (5.0)	1.6 (5.3)	<0.0001
Relative telomere length	μ (SD)	1.22 (0.31)	1.03 (0.27)	<0.0001
Frailty index (in %)	μ (SD)	25.0 (14.7)	25.5 (15.1)	0.41
Sex				
Females	*n* (%)	484 (50.0)	464 (54.5)	0.051
Males	*n* (%)	485 (50.1)	387 (45.5)	
Smoking behavior^c^				
Never	*n* (%)	455 (48.0)	371 (45.0)	0.38
Former	*n* (%)	320 (33.7)	284 (34.5)	
Current	*n* (%)	174 (18.3)	169 (20.5)	
Alcohol consumption^c^				
None	*n* (%)	300 (33.6)	260 (33.7)	0.76
<20 g/d (women), <40 g/d (men)	*n* (%)	524 (58.6)	458 (59.4)	
20+ g/d (women), 40+ g/d (men)	*n* (%)	70 (7.8)	53 (6.9)	
History of cancer^c^				
Self-report negative	*n* (%)	882 (93.2)	742 (90.5)	0.034
Self-report positive	*n* (%)	64 (6.8)	78 (9.5)	

Dataset 1, consecutively recruited subsample of the source study. Dataset 2, sampled in the context of a case-cohort study. For details, see text

^a^ Chi-square test for categorical variables, *t* test for continuous variables

^b^ Difference-based age acceleration, i.e., methylation age—chronological age

^c^ Missing values (dataset 1, dataset 2) in smoking (20, 27), alcohol consumption (75, 80), and history of cancer (23, 31)

### Age acceleration and telomere length

When exploring the sex-adjusted associations of chronological age and methylation age with relative telomere length in regression models of our combined dataset, the associations of both variables with relative telomere length were clearly significant (*p* < 0.0001; details not shown). The corresponding *F* values were 46.8 for chronological age and 32.9 for DNA methylation age, suggesting a possibly closer correlation with cellular senescence for the former variable. The results of regression analyses of relative telomere length on difference-based methylation age acceleration are shown in Table 2. In both ESTHER subsets and in the combined analysis, the estimated coefficients were small and not statistically significant. The results of inverse sampling probability-weighted regression (i.e., adjusting for the case-cohort nature of substudy 2; see the “Methods” section) were overall similar to the main analyses. For example, the estimate in the age-, sex- and leukocyte distribution-adjusted model was −0.0004 instead of −0.0006, and the result in the model additionally adjusted for cancer history was similarly stable and likewise changed to -0.0004 instead of −0.0006 (details not shown). Note that these and all other regression models in the present work included a random effect for the methylation array in order to remove potential confounding by batch effects.

**Table 2:** Results of linear mixed regression models predicting relative telomere length (RTL) from difference-based methylation age acceleration

Covariables	Dataset 1	Dataset 2	Overall	*p* ^a^
None	0.0013 (−0.0017, 0.0043)	−0.0016 (−0.0044, 0.0012)	0.0000 (−0.0021, 0.0021)	1.00
Age	−0.0009 (−0.0039, 0.0022)	−0.0031 (−0.0060,−0.0003)	−0.0018 (−0.0039, 0.0003)	0.094
Age, sex	−0.0004 (−0.0035, 0.0027)	−0.0021 (−0.0050, 0.0007)	−0.0011 (−0.0032, 0.0010)	0.30
Age, sex, leucocyte distribution (LD)	0.0001 (−0.0033, 0.0034)	−0.0014 (−0.0044, 0.0016)	−0.0006 (−0.0028, 0.0017)	0.63
Age, sex, LD, smoking	0.0001 (−0.0033, 0.0035)	−0.0015 (−0.0046, 0.0015)	−0.0006 (−0.0028, 0.0017)	0.63
Age, sex, LD, alcohol	−0.0002 (−0.0036, 0.0033)	−0.0017 (−0.0048, 0.0015)	−0.0009 (−0.0032, 0.0015)	0.48
Age, sex, LD, history of cancer	0.0001 (−0.0032, 0.0035)	−0.0017 (−0.0047, 0.0013)	−0.0006 (−0.0029, 0.0016)	0.58
Age, sex, LD, interaction (age accel. with sex)				
Estimate in females	−0.0020 (−0.0064, 0.0023)	−0.0014 (−0.0054, 0.0026)	−0.0011 (−0.0041, 0.0018)	0.45
Estimate in males	0.0024 (−0.0022, 0.0070)	−0.0014 (−0.0056, 0.0028)	0.0001 (−0.0030, 0.0032)	0.96

Shown is the estimated change (95 % confidence interval) in RTL per year of age acceleration. All models are adjusted for methylation array batch and telomere assay batch using a random effect

^a^ *p* values refer to type 3 tests of fixed effects of the overall model

### Age acceleration and frailty

The regression modeling of the frailty index on epigenetic age acceleration is summarized in Table 3. The estimates suggested positive associations between age acceleration and frailty index (FI) in both datasets, with statistically significant results in the fully adjusted models in both individual datasets and the combined analysis. After adjustment for age, sex, and leukocyte distribution, FI increased by about 0.25 % points per year of epigenetic age acceleration.

**Table 3:** Results of linear mixed regression models predicting the frailty index (FI) from difference-based methylation age acceleration

Covariables	Dataset 1	Dataset 2	Overall	*p* ^a^
None	−0.023 (−0.208, 0.162)	0.087 (−0.107, 0.281)	0.039 (−0.092, 0.170)	0.56
Age	0.167 (−0.019, 0.353)	0.214 ( 0.021, 0.407)	0.183 ( 0.053, 0.313)	0.0059
Age, sex	0.183 (−0.005, 0.371)	0.242 ( 0.046, 0.439)	0.201 ( 0.069, 0.333)	0.0028
Age, sex, leukocyte distribution (LD)	0.250 ( 0.047, 0.453)	0.274 ( 0.068, 0.481)	0.255 ( 0.115, 0.396)	0.0004
Age, sex, LD, smoking	0.243 (0.038, 0.448)	0.282 (0.074, 0.491)	0.256 (0.113, 0.398)	0.0004
Age, sex, LD, alcohol	0.289 (0.080, 0.497)	0.280 (0.070, 0.490)	0.277 (0.133, 0.421)	0.0002
Age, sex, LD, history of cancer	0.234 (0.030, 0.437)	0.275 (0.067, 0.484)	0.250 (0.108, 0.391)	0.0006
Age, sex, LD, interaction (age accel. with sex)				
Estimate in females	0.304 ( 0.036, 0.572)	0.246 (−0.026, 0.519)	0.269 (0.082, 0.455)	0.0048
Estimate in males	0.190 (−0.089, 0.469)	0.307 ( 0.013, 0.601)	0.241 (0.046, 0.436)	0.016

Shown is the estimated change (95 % confidence interval) in FI (expressed in %) per year of age acceleration. All models are adjusted for the methylation array batch using a random effect

^a^ *p* values refer to *t* distribution tests of the estimates obtained by multiple imputation in the overall model

To improve the interpretability of this result, we aimed to derive a more tangible presentation of the estimated association. Since the FI used increases by about 2.9 % points per deficit, a 0.25 % points increase per year of epigenetic age acceleration translates into one additional deficit per 2.9/0.25 = 11.6 years of acceleration. Rounding conservatively, we thus may state that our results suggest one added deficit per 12 years of methylation age acceleration, or half an added deficit per 6 years of methylation age acceleration.

The adjustment for additional variables had no relevant impact, and the results were comparable when analyzing women and men separately. In the latter analysis, the confidence intervals around the male estimate in dataset 1 and around the female estimate in dataset 2 included the null effect, which might be due to sample size limitations. Weighted regression likewise produced similar results (details not shown).

### Interaction analysis of methylation and telomeres on frailty

In the context of predicting frailty from relative telomere length, the additional consideration of an interaction with epigenetic age acceleration did not improve the prediction of FI (Table 4), which renders it unlikely that differences in epigenetic age acceleration could be responsible for the inconsistency of findings on associations between FI and telomere length in previous studies (see the “Discussion” section below).

**Table 4:** Linear regression models predicting frailty from relative telomere length (RTL) within tertiles of difference-based methylation age acceleration

Stratum of age acceleration	Dataset 1	Dataset 2	Overall	*p* ^a^
Tertile 1 (below −1.85 years)	0.132 (−1.253, 1.517)	−0.809 (−3.548, 1.930)	−0.106 (−1.274, 1.063)	0.86
Tertile 2 (−1.85 to <2.43 years)	−0.982 (−2.635, 0.672)	−0.404 (−2.266, 1.459)	−0.506 (−1.689, 0.678)	0.40
Tertile 3 (≥2.43 years)	0.960 (−0.763, 2.682)	−0.135 (−1.881, 1.611)	0.338 (−0.829, 1.504)	0.57

Shown is the estimated change (95 % confidence interval) of the frailty index (expressed in %) per standard deviation of RTL. All models adjusted for age, sex, leukocyte distribution, and random effects of telomere and methylation array batch

^a^ *p* values refer to *t* distribution tests of the estimates obtained by multiple imputation in the overall model

## Discussion

In this study of more than 1800 community-dwelling adults, there was evidence for an independent association of epigenetic age acceleration with frailty as measured by a deficit accumulation-based approach. Quantitatively, the observed association translated into round about half an additional deficit per 6 years—i.e., roughly 1.2 standard deviations—of age acceleration. Relative telomere length, on the other hand, was not significantly associated with age acceleration. These findings suggest that DNAm age acceleration might be correlated with clinically relevant aging-related phenotypes, in particular frailty, due to pathways unrelated to genomic age as assessed by TL.

### Age acceleration is associated with a comprehensive frailty measure

The potential relationship between epigenetic age acceleration and frailty-related phenotypes apparently has been investigated only in one previous study: in an analysis of the Lothian Birth Cohort 1936 (LBC1936), significant correlation coefficients ranging from −0.05 to −0.07 were found between DNAm age acceleration and cognitive functioning, grip strength, or lung function [10].

Given that the LBC1936 participants were rather strictly 70 years of age when assessed for the study of age acceleration, the present findings extend the prior evidence from the old to middle-aged-old age group, as they were based on study participants aged 50 to 75 years. Moreover, whereas age acceleration was successfully analyzed with respect to three individual healthy aging-related characteristics in LBC1936, the current work employed the frailty index, a more multi-dimensional approach that combines parameters of multiple physiological systems and functional capacities [4]. This very robust frailty measure features strong replicability and validity across populations and datasets [13], fostering the relevance of the present findings and supporting a wide applicability to frailty-related research questions including study populations that may lack information on one frailty item or another.

Additional explorations of the association of DNAm age acceleration with subcomponents of our frailty index, which had been suggested by a reviewer, yielded intriguing additional insights: using z-transformed variables in order to obtain comparable association estimates, we found DNAm age acceleration to be associated with the 1-item self-rated general health subcomponent (estimated coefficient, 0.016; *p* = 0.0015), the 11-item disease history subcomponent (0.015, *p* = 0.0014), and the 16-item activities of daily living subcomponent (0.015, *p* = 0.0019), but not with the 6-item symptoms subcomponent (0.009, *p* = 0.061), based on the overall model adjusted for age, sex, and leukocyte distribution. The first three association estimates were rather close to the corresponding value of 0.017 pertaining to the z-standardized full frailty index. These findings generally support the robustness of the association of epigenetic age acceleration with frailty across diverse domains, whereas the null finding for the symptoms subcomponent might reflect its substantial inherent subjectiveness.

Epigenetic processes have been termed an “attractive candidate” for explaining frailty differences, and methylation levels of some promoter CpG islands are associated with frailty [14]. Findings for global DNA methylation and frailty are somewhat inconsistent, though this might be due to methodological differences [7, 14]. Major hypotheses advanced in this context include that the activation of genes involved in a response to frailty leads to a hypomethylation of respective regulatory genomic regions or that deficits in methylation maintenance lead to a dysregulation of gene expression and the development of frailty [7]. Intriguingly, the LBC1936 study found epigenetic age acceleration but not individual CpG methylation levels to be associated with their fitness measures [10], suggesting that an “accelerated” epigenetic aging may be more closely correlated to clinically relevant frailty phenotypes than any individual CpG. However, it ultimately remains unclear how the interplay of environmental factors and stochastic processes leads to the manifestation of a consistent (and possibly mechanistically relevant) epigenetic clock at specific loci in contrast to an overall inconsistent (and purely correlational) epigenetic drift [15].

### Age acceleration and telomere length are not correlated

In the absence of previous pertinent publications, the analysis of DNAm age acceleration with TL was motivated by the hypothesis that accelerated epigenetic aging could plausibly be associated also with cellular senescence. The ESTHER study provided no evidence for such an association. It was furthermore hypothesized that differences in DNAm age acceleration might be responsible for the inconclusive prior reports about an association of TL with frailty-related phenotypes [8, 9, 12]. However, no association of TL with frailty was found in the ESTHER cohort, regardless of the level of DNAm age acceleration.

A limited number of studies have investigated DNA methylation in association with telomere length. Global hypomethylation has been suggested to be associated with decreasing TL [16], and several individual CpGs are correlated with TL independent of chronological age [17]. Age-related differences in the methylation of subtelomeric regions further support a close link of epigenetics and cellular senescence [18]. The absence of an association of TL with epigenetic age acceleration, however, seems to be in line with evidence suggesting that DNAm age does not reflect mitotic age [2], which is a major determinant of age-dependent telomere shortening [19].

### Limitations and strengths

Given the observational, cross-sectional design of the present study, our findings should not be interpreted as reflecting causality. Future studies should consider repeated measurements of methylation/age acceleration as well as telomere length and frailty in order to approach this issue. Methylation analyses were done on whole-blood DNA, which constitutes a mixture of cells present in the peripheral circulation. Although methylation patterns are known to vary between tissues, DNAm age as used in the present study’s main analyses features only a low correlation with cell types, presumably because it has been consciously designed as a multi-tissue predictor based on rather diverse learning data sets [2, 11]. Differential blood counts were not available in the present study, but our main models were adjusted for leukocyte subtype distributions estimated by the Houseman method, and this had only a minor impact on the results. Contrasting the aforementioned limitations, the large size and representative nature of the study sample, as well as the use of a thoroughly constructed multi-dimensional frailty measure, are major strengths of the present work, which featured an altogether exceptional combination of data on methylation, telomeres, and frailty.

### Towards a better understanding of the human clocks

Knowledge on the interplay of genomic, epigenetic, and bioclinical aging phenomena remains surprisingly vague. Even though telomere length has been suggested to have some effects on phenotype development, current evidence altogether seems to emphasize its role as a rather innocent bystander of aging and an indicator of lifetime exposures [19]. Aging-associated CpG sites also are not generally related to known mechanisms of aging [20], and it has been described as one of the chief challenges in this field “to identify the most important genes and pathways for which altered methylation patterns contribute to age-related functional decline” [21]. Our study contributes to an increasing body of literature that suggests that the epigenetic clock relates to a molecular process that might play a causative role in biological aging, as exemplified by recent reports showing that epigenetic age acceleration is prognostic of all-cause mortality [22] and is increased in Down syndrome, which is a segmental progeria [23]. However, the mechanistically causal nature of *any* altered DNA methylation patterns—including epigenetic age acceleration—for aging-related phenotypes at present remains speculative. The most promising way to address this issue may be genomewide association studies of frailty-associated differential CpG methylation and DNAm acceleration, which could yield instrumental variables for use in so-called Mendelian randomization studies, an analytical approach specifically designed for studying causality in observational settings [24].

## Conclusions

Confirming speculations by Horvath, the present findings suggest that epigenetic aging contains information complementary to that of the telomere clock [2]. The results on epigenetic age acceleration being associated with a multi-dimensional frailty phenotype appear promising, but innovative approaches like Mendelian randomization will be needed to elucidate the causal relevance of such patterns linking epigenetic, genomic, and clinical correlates of age.

## Methods

### Study design and study population

The present study was based on the ESTHER epidemiological cohort study, which is an observational study of the elderly general population of Saarland, a federal state of Germany [25]. In brief, almost 10,000 participants aged 50 to 75 years were recruited by their general practitioner when presenting for routine health checkups from June 2000 to December 2002. This cohort is representative for this age segment of the community-dwelling general population of Saarland [25].

In brief, the baseline assessment forming the basis of the present investigation consisted of obtaining the data collected as part of the health checkup, drawing a blood sample that was mailed to the study center and stored at −80 °C until analysis, and completing a detailed standardized questionnaire on socio-demographics, lifestyle factors, and medical history.

For the present study, only subjects with data available on epigenetic age, telomere length, and frailty (see below) were considered, and the availability of DNA methylation data was the principal limiting factor in this regard. DNA methylation measurements allowing the calculation of epigenetic age had been obtained from two subsamples of the source study: substudy 1 included 1000 consecutively recruited ESTHER participants with sufficient baseline DNA available; substudy 2 included all ESTHER participants deceased until year 8 follow-up and with sufficient DNA available (*n* = 406 after discounting 196 individuals already included in substudy 1), plus 458 additional subjects randomly selected from ESTHER participants with sufficient DNA available and not included in substudy 1 or the deceased group. After discounting subjects with missing data on telomere length, a total of 1820 participants could be included in the present analysis (969 (97 %) of substudy 1; 851 (98 %) of substudy 2).

### Ethics, consent, and permissions

Inclusion in the ESTHER study, which complies with the Declaration of Helsinki, was conditional upon written informed consent. The study protocol and procedures were approved by the ethics committees of both the Medical Faculty of the University of Heidelberg and of the Medical Association of Saarland.

### Epigenetic age and age acceleration

The DNAm age was calculated based on a predictor developed in a large study of genomewide methylation array datasets [2] using the R tutorial of the pertinent publication. Note that DNAm age was calculated using the exact predictor developed by Horvath [2] without re-training the model on the present data. In brief, DNAm age is calculated from methylation levels at 353 CpG sites. This measure has been suggested to reflect the “cumulative work done by an epigenetic maintenance system” and is highly correlated with chronological age [2]. The so-called difference-based DNAm age acceleration can be calculated by subtracting the chronological age from the predicted DNAm age [2].

Methylation levels were determined using the Infinium HumanMethylation450 BeadChip (Illumina, San Diego, CA) at the Genomics and Proteomics Core Facility of the German Cancer Research Center, Heidelberg, Germany. Methodological details have been published previously [1].

### Telomere length

As a measure of relative telomere length (TL), the telomere repeat copy number to the number of single-copy gene ratio (T/S ratio) was determined using a quantitative PCR approach [26]. The assay used the single-copy gene *36B4* for reference, and the PCR was done on a Lightcycler® 480 (Roche Diagnostics, Mannheim, Germany). Further details on the TL measurements, including quality control and assay validation, have been published elsewhere [27].

### Frailty index

As a measure of frailty, a FI based on the accumulation of deficits was calculated as previously described [3]. In brief, the FI is defined as the proportion of deficits present, where the exact deficits considered in the construction of the index depend on the available data [13]. In the ESTHER study population, the FI was constructed following standard recommendations [13] and ultimately based on 34 deficits (i.e., one additional deficit increases this FI by 0.029 or 2.9 % points), including poor self-rated general health, history of various diseases (11 items: myocardial infarction, angina pectoris, heart failure, stroke, hypertension, hyperlipidemia, diabetes, cataract, glaucoma, gout, cancer), difficulties in the activities of daily living (16 items: “vigorous activities,” “climbing several flights of stairs,” “climbing one flight of stairs,” “walking more than one mile,” “walking several blocks,” “walking one block,” “moderate activities, such as moving a table, pushing a vacuum cleaner, bowling, or playing golf,” “lifting or carrying groceries,” “bathing or dressing yourself,” “bending, kneeling or stooping,” “limits in normal work or activities due to pain,” “accomplished less work or activities due to impaired physical health,” “limits in type of work or activities due to impaired physical health,” “difficulties chewing hard food,” “difficulties chewing meat,” “short-term memory loss”), and various symptoms (six items: under-/overweight, pyrosis, shiver, insomnia, costiveness, aconuresis). Missing values in the variables needed for the frailty index calculation were dealt with by multiple imputation [3], and models including FI in the present analysis were based on 20 imputations combined by the SAS procedure MIANALYZE.

### Statistical methods

The study population was first described with respect to the main analysis variables, major participant characteristics, and important covariables (smoking behavior (never, former, current), alcohol consumption (none, 1–19 (women) or 1–39 (men) g/d, 20+ (women) and 40+ (men) g/d), history of cancer). Histograms were used to explore the distribution of DNAm age acceleration, TL, and FI. Subsequently, linear regression models predicting TL or FI from DNAm age acceleration were fitted with increasing adjustment sets: no covariables; age; age, sex; age, sex, and leukocyte subtype distributions (LD) estimated according to the Houseman method [28] (main model). Random effects were included in the models to account for methylation array and telomere assay batch effects. Subsequently, the sensitivity of the main model results to additional adjustment for smoking, alcohol or history of cancer was studied. In addition, sex-specific estimates were examined. Finally, the potential interaction of telomere length and epigenetic age acceleration on frailty was examined by fitting linear regression models predicting FI from TL within tertiles of DNAm age acceleration. For this purpose, linear models including continuous TL, DNAm age acceleration tertiles, and the interaction of these two variables were analyzed; the categorization and way of presentation as nested effects was chosen for the ease of interpretability.

The regression models were generally fit first to dataset 1 and dataset 2 separately and then to the combined dataset. Additional sensitivity analyses included the use of inverse sampling probability weights (i.e., adjustment for the oversampling of deceased subjects due to the case-cohort design of substudy 2) in the combined dataset models. Statistical significance was defined as *p* < 0.05. All data analyses were done using SAS 9.3.

## Availability of data and materials

Data protection standards and assurances made as part of the informed consent procedure of ESTHER preclude the publication of the source data in publicly available repositories. However, individual data access may be granted within a framework of scientific cooperation.

## Additional file

Additional file 1: Figure S1. Distribution of main analysis variables. Shown are the histograms (overlay: fitted Normal distribution) of difference-based DNAm age acceleration (A, B), relative telomere length (C, D), and frailty index (E, F; based on one instance of the multiple imputation procedure). Left plots refer to dataset 1 (consecutively recruited subsample of source study; *n* = 969), right plots to dataset 2 (case-cohort subsample of source study; *n* = 851). (PDF 1818 kb)
