# Supplementary material for: Frailty is associated with the epigenetic clock but not with telomere length in a German cohort
Source: Clin Epigenetics. 2016 Feb 26;8:21. doi: 10.1186/s13148-016-0186-5 (PMC4768341; doi:10.1186/s13148-016-0186-5)

Additional file 1: Figure S1. Distribution of main analysis variables. Shown are the histograms (overlay: fitted Normal distribution) of difference-based DNAm age acceleration (A, B), relative telomere length (C, D), and frailty index (E, F; based on one instance of the multiple imputation procedure). Left plots refer to dataset 1 (consecutively recruited subsample of source study; n=969), right plots to dataset 2 (case-cohort subsample of source study; n=851).

A

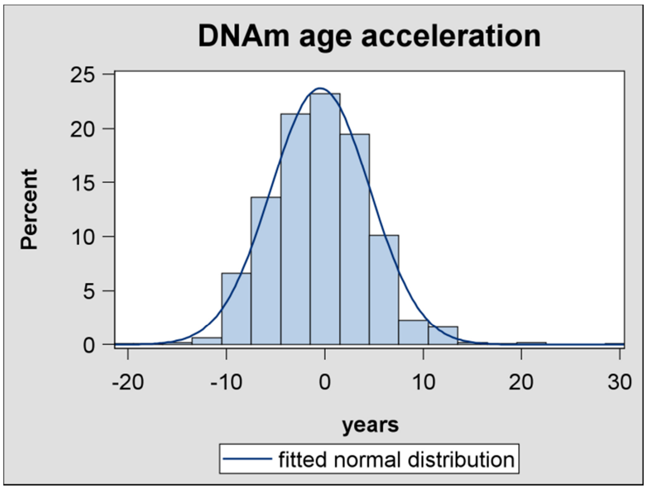

B

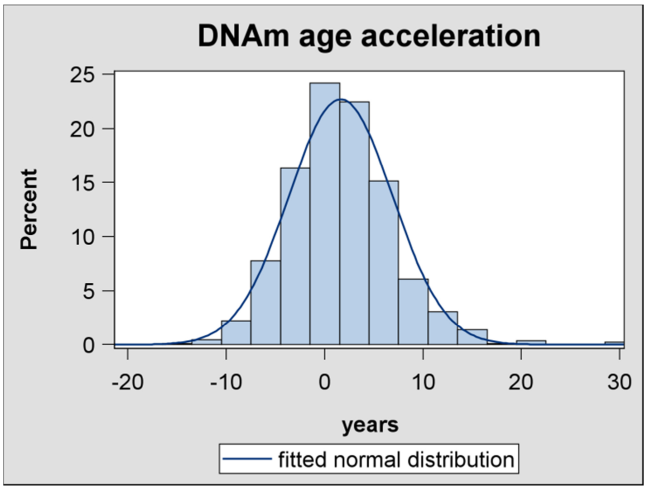

C

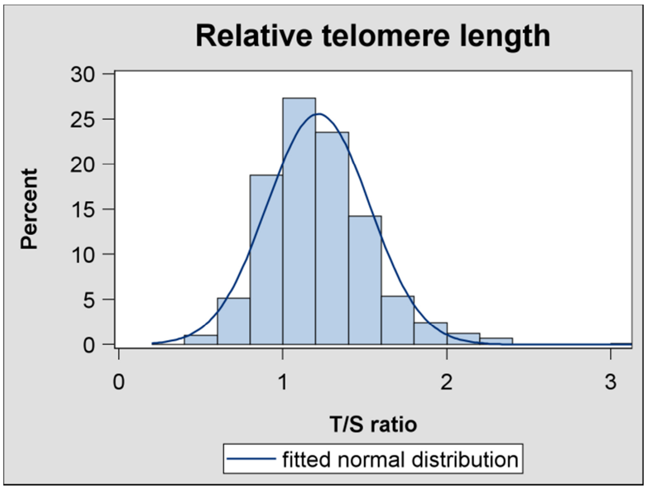

D

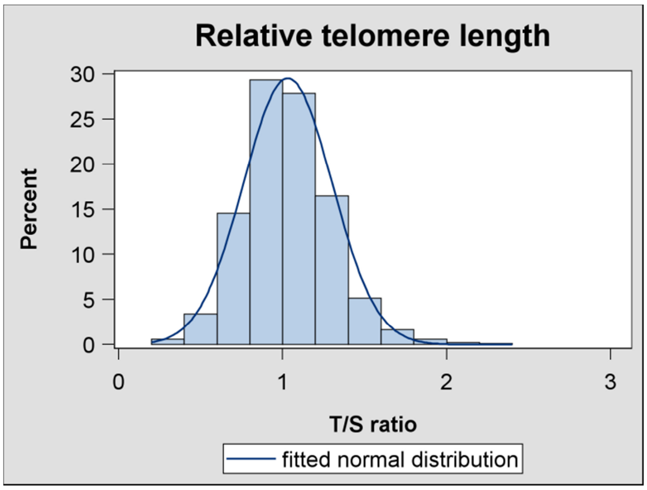

E

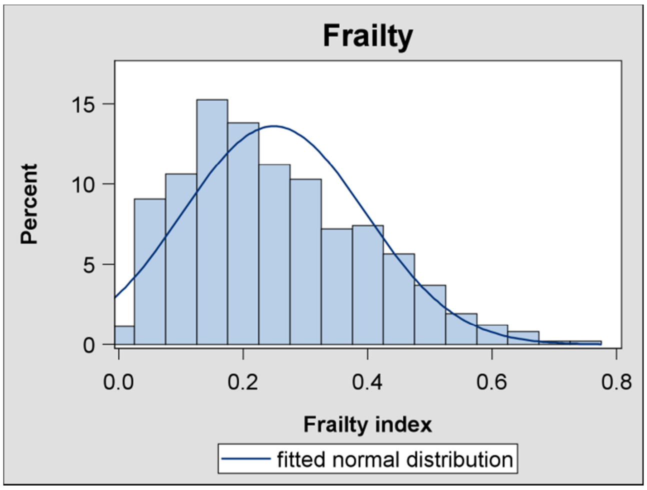

F

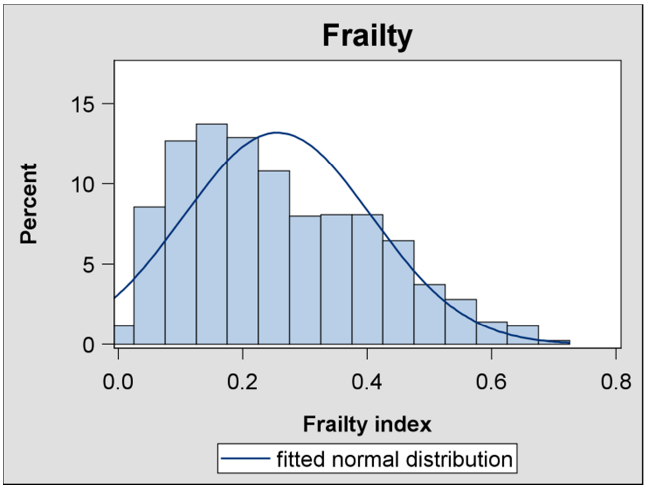

Supplement: Additional file 1: Figure S1. — Distribution of main analysis variables. Shown are the histograms (overlay: fitted Normal distribution) of difference-based DNAm age acceleration (A, B), relative telomere length (C, D), and frailty index (E, F; based on one instance of the multiple imputation procedure). Left plots refer to dataset 1 (consecutively recruited subsample of source study; n = 969), right plots to dataset 2 (case-cohort subsample of source study; n = 851). (PDF 1818 kb) [file 13148_2016_186_MOESM1_ESM.pdf]
